# A double blind community-based randomized trial of amoxicillin versus placebo for fast breathing pneumonia in children aged 2-59 months in Karachi, Pakistan (RETAPP)

**DOI:** 10.1186/s12879-015-1334-9

**Published:** 2016-01-13

**Authors:** Fyezah Jehan, Muhammad Imran Nisar, Salima Kerai, Nick Brown, Benazir Balouch, Zulfiqar Hyder, Gwen Ambler, Amy Sarah Ginsburg, Anita K. M. Zaidi

**Affiliations:** 1 Department of Paediatrics and Child Health, Aga Khan University, Stadium Road, PO Box 3500, Karachi, 74800 Pakistan; 2 Salisbury District Hospital Foundation Trust, Salisbury, Wiltshire UK; 3 PATH, Seattle, Washington USA

**Keywords:** Fast breathing pneumonia, Pneumonia, Integrated management of childhood illnesses, Amoxicillin, Placebo

## Abstract

**Background:**

Fast breathing pneumonia is characterized by tachypnoea in the absence of danger signs and is mostly viral in etiology. Current guidelines recommend antibiotic therapy for all children with fast breathing pneumonia in resource limited settings, presuming that most pneumonia is bacterial. High quality clinical trial evidence to challenge or support the continued use of antibiotics, as recommended by the World Health Organization is lacking.

**Methods/design:**

This is a randomized double blinded placebo-controlled non-inferiority trial using parallel assignment with 1:1 allocation ratio, to be conducted in low income squatter settlements of urban Karachi, Pakistan. Children 2–59 months old with fast breathing, without any WHO-defined danger signs and seeking care at the primary health care center are randomized to receive either three days of placebo or amoxicillin. From prior studies, a sample size of 2430 children is required over a period of 28 months. Primary outcome is the difference in cumulative treatment failure between the two groups, defined as a new clinical sign based on preset definitions indicating illness progression or mortality and confirmed by two independent primary health care physicians on day 0, 1, 2 or 3 of therapy. Secondary outcomes include relapse measured between days 5–14. Modified per protocol analysis comparing hazards of treatment failure with 95 % confidence intervals in the placebo arm with hazards in the amoxicillin arm will be done.

**Discussion:**

This study will provide evidence to support or refute the use of antibiotics for fast breathing pneumonia paving a way for guideline change.

**Trial registration:**

Clinical Trials (NIH) Register NCT02372461

## Background

World Health Organization (WHO)-defined pneumonia, a spectrum of clinical signs and symptoms varying from isolated fast breathing to presence of danger signs, is an important cause of child morbidity and mortality in low resource countries [1]. As a result, the WHO has recommended antibiotic prescription guidelines to cover the probability that most children with pneumonia-like symptoms have bacterial infections [2]. Recently, this broad recommendation for empiric antibiotic use in all children with pneumonia, including those with isolated fast breathing, has been questioned [3–7]. Furthermore, with the advent of *Haemophilus Influenza* type b (Hib) and pneumococcal vaccine in the Expanded Program of Immunization, there is protection against two most common bacterial pathogens of bacterial pneumonia. This has resulted in far smaller proportions of children with LRTI having a bacterial etiology and, as a corollary, more having benign viral infections [1, 8, 9]. In Pakistan for example, the Hib vaccine was introduced in the universal immunization program in 2009 and pneumococcal conjugate vaccine in 2013-14. Vaccine study for three doses of Hib has demonstrated the vaccine effectiveness in reduction of pneumonia from 62 %–70 % among Pakistani children [10], and pneumococcal vaccine studies are currently underway.

Children with ‘fast breathing pneumonia’ (previously classified by the WHO as “non-severe pneumonia”) relatively have mild illness, which in up to 65 % of cases is viral (8) with many of the remainder having self-limiting infection [11]. Spontaneous remissions are common and may render antibiotics redundant and potentially harmful. The risk of harm from unnecessary antibiotic use is twofold: individual and population level. At the individual level, side effects to antibiotics, both unpleasant and dangerous, must be considered. Frequent exposure to antibiotics has also been shown to have long-term deleterious effects on the native gut micro-biota that may impair growth and nutrition in children [12]. As a result of this, there may be altered immune processing resulting in long- term risk of subsequent infections [13–15]. At a public health population-based level, there is the risk of potential emergence of resistance of common pathogens to first line antibiotics and the need to use more expensive (sometimes more toxic) alternatives [16–18]. Infections due to antibiotic resistant pathogens not only make people ill for longer but also unnecessarily burden health care resources and increase cost [19].

The cost of antibiotic treatment for all children with WHO-defined pneumonia is formidable. An estimated US$ 200 million is the cost of antibiotic treatment for all children with pneumonia in South Asia & sub Saharan Africa [20]. In Pakistan, the average cost to treat one episode of pneumonia in a child as an outpatient was estimated to be US$ 13.44 in 2006, representing 82 % of annual health expenditure per person at that time [21]. Unnecessary antibiotic prescription is a major contributor to antibiotic resistance and puts a strain on individuals and under-resourced program in low-income countries. This crisis has arisen largely as a result of increased antibiotic use with the predictable consequence of microbial mutation to the point that, in some settings, many previously pan-susceptible organisms are now almost untreatable. The problem is particularly acute in South Asia. A recent systematic review of etiology in developing countries of infant sepsis aged 1–12 months with no clear focus of infection in developing countries has shown in vitro susceptibility to chloramphenicol/penicillin, penicillin/gentamicin, and third-generation cephalosporin of only 47 %, 63 %, and 64 %, respectively [22].

The last comprehensive Cochrane review looking at use of antibiotics in pneumonia conducted up to 2009 showed that among 27 studies, comparing multiple antibiotics, none were found to compare antibiotic with placebo [23]. Another Cochrane review done last year which looked at antibiotic versus no antibiotic therapy in children with non-severe pneumonia and wheeze did not find a single study meeting inclusion criteria and concluded that there is a lack of evidence and randomized controlled trials are needed to address this question in representative population [24]. Two recent randomized controlled trials (RCTs) compared placebo to amoxicillin for the management of non-severe pneumonia, now called fast breathing pneumonia. Hazir et al [5] conducted a double-blinded, RCT of oral amoxicillin versus placebo for non-severe pneumonia in four centers in Pakistan, using age-dependent WHO cut-offs for dosing. Cumulative treatment failure (TF) by day 3, defined as appearance of any danger sign or onset of lower chest indrawing, was similar in both groups: 7.2 % (31/431) of children in the amoxicillin group and 8.3 % (37/442) in the placebo group. Between the arms there was no statistically significant difference in relapse by day 14, the number of children requiring change (or initiation) of antibiotics, the number of children who developed danger signs, or the number of children requiring hospitalization. There were no deaths. The second randomized placebo controlled multi-centre trial was conducted in India in out-patient clinics associated with tertiary care hospitals [4]. This trial used the WHO criteria for non-severe pneumonia, but only enrolled children with wheeze (audible or auscultatory). TF rates at day 4 was defined as development of WHO-defined severe or very severe pneumonia, hypoxaemia (SpO_2_ < 90 %), fever (temperature >101^0^ F) or persistence of non-severe pneumonia, or wheeze. TF among placebo recipients was 24.0 % (201/836), while among the amoxicillin group it was 19.9 % (166/835). The rate difference was 4.2 % (95 % CI 0.2 to 8.2); however, there was no difference in the rates of relapse. Clinical failure due to the development of severe or very severe pneumonia or hypoxemia was similar between groups. These trials were conducted before Hib and pneumococcal vaccines were introduced in the local setting.

In summary, there is equipoise regarding utility of antibiotics for mild lower respiratory tract illnesses (LRTI). In children 2 to 59 months of age there is no high quality trial evidence in managing children with fast breathing pneumonia in community settings and the WHO has called for evidence on which to update guidelines [25]. Our proposed trial is comparing standard antibiotic treatment with placebo for children 2 to 59 months of age in poor urban slum settings in Karachi, Pakistan where pneumococcal or Hib vaccine has been previously implemented.

### Hypothesis

The null hypothesis for this trial is that the absolute treatment failure rate of children 2–59 months of age presenting at a primary healthcare (PHC) facility for treatment of WHO defined fast breathing pneumonia who receive 3 days of placebo is worse than 3 days of amoxicillin by 2.5 % (TF_placebo_—TF_amoxicillin_ > 2.5 %, assuming TF of 5 % in amoxicillin group).

The alternate hypothesis is that absolute treatment failure rate of children 2–59 months of age presenting at a primary healthcare (PHC) facility for treatment of WHO defined fast breathing pneumonia who receive 3 days of placebo is equal to or not worse than 3 days of amoxicillin by 2.5 % (TF_placebo_—TF_amoxicillin_ ≤ 2.5 % assuming TF of 5 % in amoxicillin group).

### Research question

The primary research question is to assess among children 2 to 59 months of age with WHO-defined fast breathing pneumonia presenting at a PHC facility, if treatment with 3 days of placebo is non-inferior to three days of oral amoxicillin (reference therapy). The secondary research question is to evaluate the proportion of children with fast breathing pneumonia who have pneumococcal carriage, viral carriage or both in nasopharyngeal specimens and to evaluate predictors of treatment failure (TF) with regards to viral or bacterial nasopharyngeal carriage.

## Methods

### Design

This is a randomized double blinded placebo-controlled non-inferiority trial with parallel assignment and treatment allocation ratio of 1:1.

### Patients

#### Setting

**Fig. 1:**
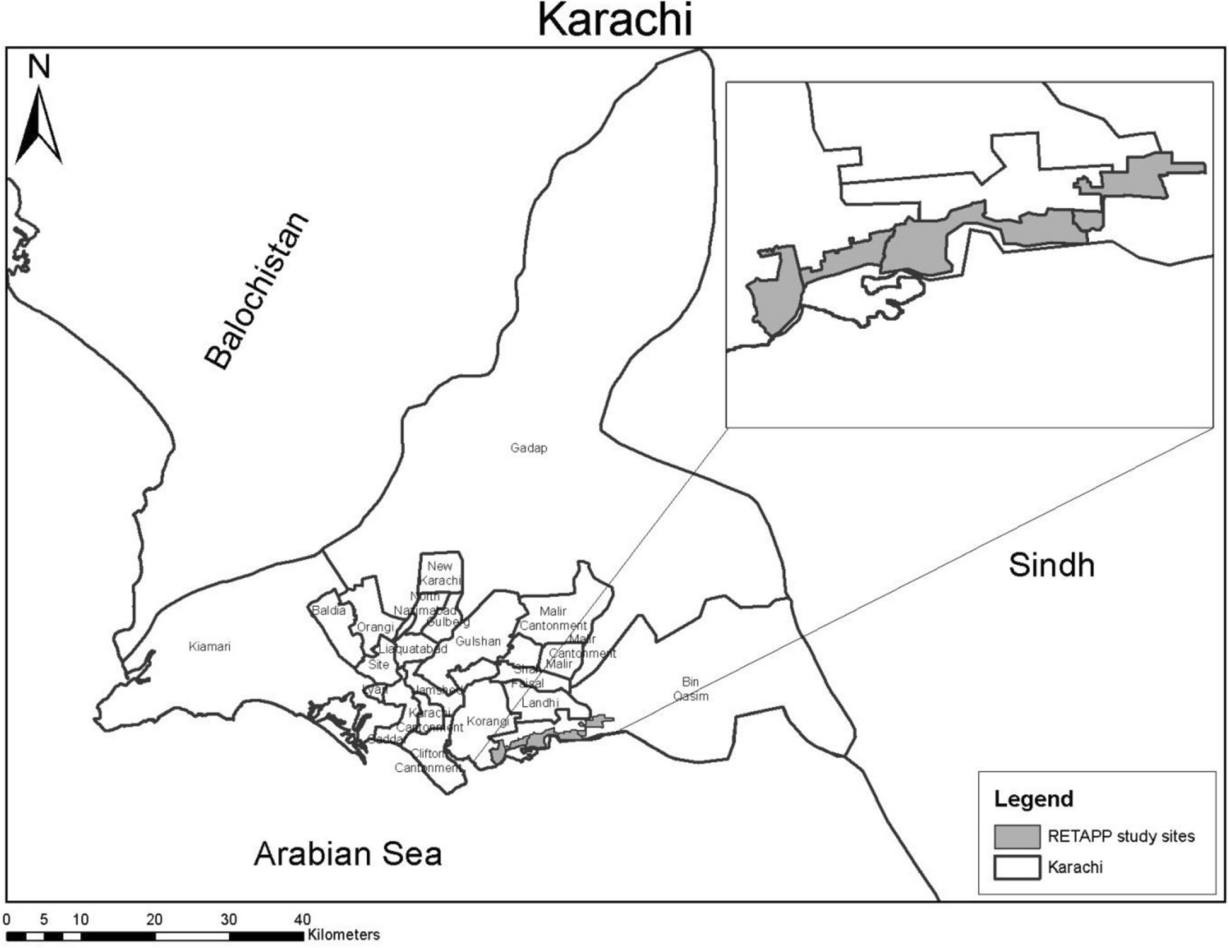
Map of Karachi and RETAPP study sites (inset). Source: Data Management Unit, Department of Pediatrics and Child Health Aga Khan University

The study is currently being carried out at four primary healthcare centers (PHC) located in low-income communities in peri-urban areas of Karachi (Fig. 1). The Department of Paediatrics and Child Health, AKU has maintained health and demographic surveillance in these areas since 2003. As of 2010 baseline census, the total population is 198,494, total children under 5 are 28,005, under-5 mortality is 76/1000 live births, HIV sero-prevalence is <0.1 % among pregnant women, and malaria endemicity is low at these sites.

#### Inclusion and exclusion criteria

Children 2–59 months old, visiting primary health clinics (PHC) run by Aga Khan University (AKU) with following eligibility criteria are included (Table 1).

**Table 1:** Eligibility Criteria

Inclusion	Exclusion
Children 2–59 months old who are visiting PHC History of cough or difficult breathing less than 14 days^a^ (observed or reported) Respiratory rate ≥ 50 breaths per minute in children 2 to <12 months OR respiratory rate ≥ 40 breaths per minute in children ≥12 months^b^ Providing written informed consent	Antibiotics taken in last 48 h Lower chest wall in-drawing Any general danger sign as defined by WHO *(Stridor when calm, hypoxia defined as SaO*_*2*_ *< 90 % in air, inability to feed, persistent vomiting, convulsions or reduced conscious level)* Bulging fontanel Pedal edema Known asthmatics, tuberculosis or other severe illness History of hospitalization in last two weeks Congenital heart disease Any surgical condition requiring hospitalization Out of catchment area Enrolled in another trial or previously enrolled in study

^a^Children, who present with wheeze along with cough or difficulty in breathing, are given trial of nebulization with bronchodilator up to three times 15–20 min apart. These children are then re-assessed for respiratory rate after each nebulization therapy. If respiratory rate remains persistently above cut-off, irrespective of wheeze, child is considered for inclusion. For persistent wheezers, oral bronchodilator is given for three days

^b^On two consecutive readings by community health worker and physicians

### Intervention

Children in the reference arm receive the WHO regimen of oral Amoxicillin in two divided doses for three days using WHO Integrated Management of Childhood Illnesses (IMCI) recommended weight bands [26] (Table 2). Children in the comparison arm receive pharmacologically inert placebo similar in appearance in two divided doses for three days.

**Table 2:** WHO age and weight bands and drug doses

Age (Weight)	Amoxicillin Dose^a^ (250/5 ml) (Morning & Evening)
2 months up to 12 months (4-˂10 kg)	5 ml
12 months up to 3 years (10- ˂14 kg)	10 ml
3 years up to 5 years (14 – ˂ 20 kg)	15 ml

^a^Or an equal amount of placebo

### Recruitment and follow-up

Children between 2 to 59 months old with cough or difficult breathing, presenting at the participating PHCs are screened for eligibility by the study physicians. If eligible the parent/guardian is referred to an independent physician to obtain written informed consent. If consent is given and child is enrolled then randomization serial identification number will be assigned. All enrolled children will receive study drug by CHW in accordance with the randomization number in morning and evening on day 0, 1 and 2.

**Fig. 2:**
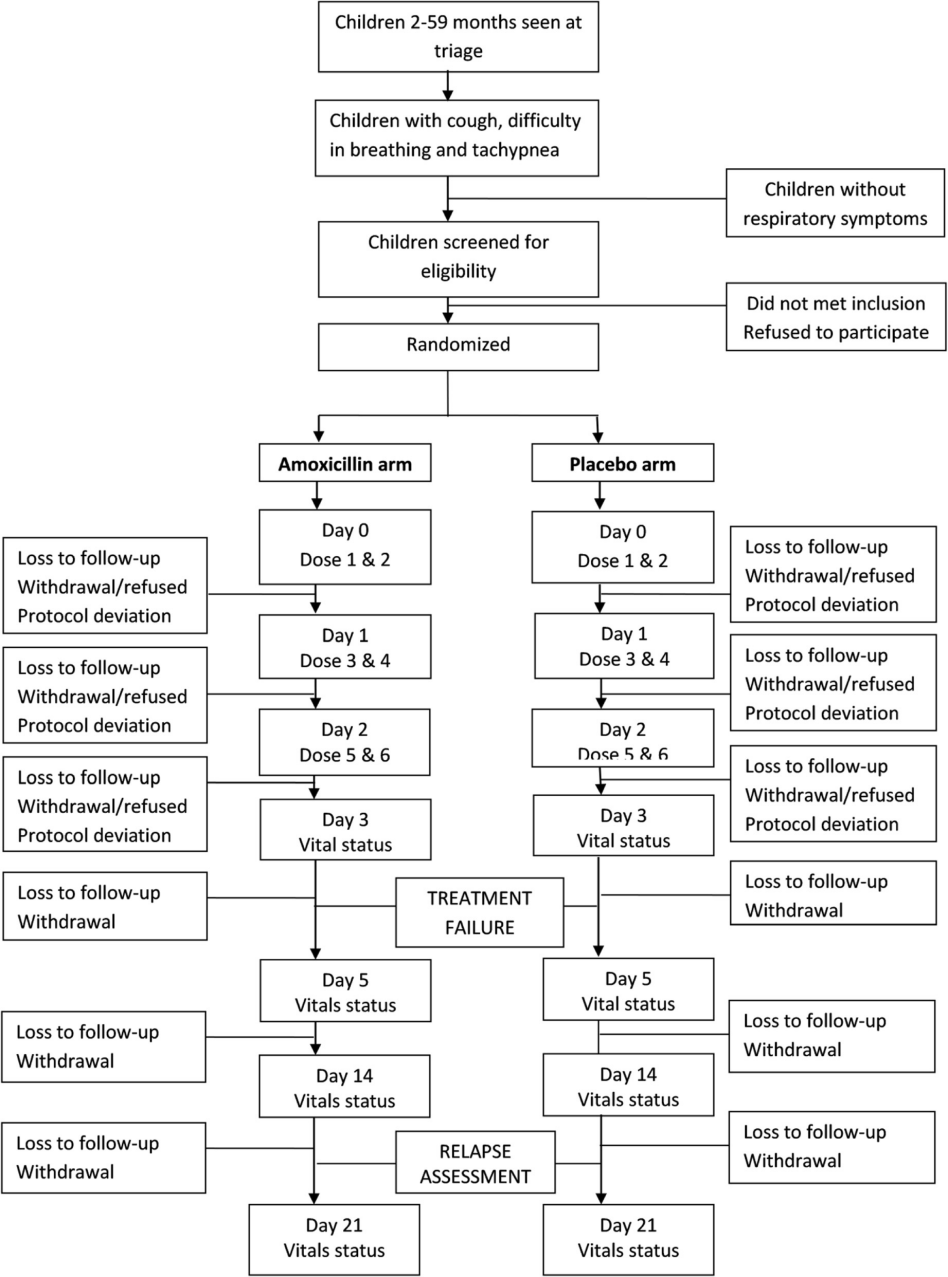
Flow of participants in the study

Children are followed for compliance and outcome twice daily on days 0, 1, 2, 3, with a morning assessment by a study physician in the study clinic and a home visit in the evening by a CHW. Subsequently, children are seen by a study physician once on days 5, 14 and 21. At follow-up visits participants will be examined by study physician in the morning and CHW in the evening for resolution of high respiratory rate along with signs of treatment failure and relapse (Fig. 2). All adverse events are recorded on the adverse event reporting form (AERF). The schedule of follow-up is derived from our experience of a trial in younger infants [27] and taking into consideration safety of the enrolled children.

### Blinding and randomization

The randomization technique is stratified block randomization with varying sized blocks of 4, 6, 8 and 10 to prevent prediction of next assignment and to ensure sequence allocation concealment. Random sequence lists are stratified by age group (2–11 months and 12–59 months) to maintain similar composition of ages in both study arms. Separate randomization lists stratified by age (‘IN’ for infants 2–12 months of age and ‘CH’ for children 12–59 months of age) with drug labels (antibiotic or placebo) will be generated. This is done to reduce the chance that prescription errors will occur as investigators are using different weight bands for antibiotic dosages in these two age groups. Sequence generation was done at the AKU Clinical Trials Unit (CTU), electronically via computer using random selection method before the start of the study. This was done by an independent person unrelated to the trial.

This is a blinded study in which the drug assignment will be concealed from patients, parents, and study personnel. To ensure blinding, self-adhesive, pre-coded sticking labels with the unique identification numbers are prepared at the CTU and applied to the opaque medication bottles before providing to the study physicians. 4 digit randomization codes are used with two prefix characters for different age groups (e.g. IN0000, CH0000). Differentiation in code by age is to minimize chances of error in prescribing the wrong bottle. The codes will be kept safe with a person completely unrelated with the trial and broken only after analysis or upon the recommendation of the DSMB. This will ensure blinding of both the allocating physician as well as the one performing outcome assessment. Moreover, medication will be dispensed by community health workers who are not involved in treatment allocation and outcome assessment.

### Sample size

In the study of Hazir et al [28], cumulative TF rates at day 3 were reported to be between 4 % and 7 % in tertiary level hospital setting. Given that the currently described study is conducted in a primary care setting, investigators have estimated that the TF rate of 5 %. Assuming a TF rate of 5 % in both the arms, using one sided alpha of 0.05, power of 85 %, non-inferiority margin of 2.5 % and an expected 10 % lost to follow-up/non per protocol, the minimum total estimated sample size required is 2430. Placebo will be judged to be “no worse” than amoxicillin if the lower bound of the 95 % confidence interval lies in the allowed margin.

### Data collection and quality assurance

All staff involved in the conduct of trial is certified in Good Clinical Practice. Two dedicated research supervisors are involved with day-to-day monitoring, ensuring quality and adherence to protocols at each step of data collection from enrollment to follow up. Refresher trainings are conducted for all staff on a quarterly basis to ensure uniformity in assessment and data collection.

Detailed case report forms (CRFs) are used for recording information on eligibility, randomization, demographics, air quality, clinical history, vital status, follow-up, adverse event, protocol violation, protocol deviation, TF and relapse. Alongside documenting clinical information, video recording of child symptoms and nasopharyngeal swab collection is done after the caregiver has provided oral consent. Nasopharyngeal swabs are collected using WHO recommendations. IMCI treatment algorithms are followed for treatment of children. Blood cultures are collected for children with TF or relapse. All TFs are assessed by physician and managed in accordance with the WHO handbook of caring for sick child [29]. Case summaries are made for all participant assessed for eligibility and are discussed within the research team for quality. 10 % of the enrollments are also concurrently re-assessed by the research supervisor for quality assurance.

### Data management

At the field sites, all CRFs are checked for errors, consistency, missing or illogical values. After the initial quality check, these case files are sent to the study data management unit for data entry. Logs of CRF transfers and handling are maintained for the custody chain. All data is doubled entered using a customized SQL-based relational database management system with an audit trail. Data is assessed for logical and consistency checks on a weekly basis. Discordant entries are verified against the original CRFs. After entry, the physical forms are stored in a secured location at the data management unit. Access to data is restrictive as to maximize security and confidentiality.

### Outcomes

The primary outcome will be cumulative treatment failure at or before day 3 (Table 3). Secondary outcome includes treatment adherence, new onset infectious co-morbidity, severe and non-severe adverse event, relapse, pneumococcal and viral carriage and cost of treatment.

**Table 3:** Treatment failure criteria^a^

Criterion	Days of assessment						
	0	1	2	3	5	14	21
Death	Treatment failure				Relapse		Vital status check
Any danger sign^b^							
Onset of lower chest in drawing							
Hospitalization due to any reason							
Change of antibiotic by study physician for new onset comorbidity or serious non-fatal antibiotic associated adverse event							

^a^Chest X-ray, blood for culture is processed for all treatment failures and relapses

^b^Unconscious/lethargy, convulsions, unable to feed, stridor when calm, hypoxia (paO2 < 90 %) in air, vomits everything

### Statistical analysis

Baseline characteristics will be compared by analyzing differences in means and proportions among the study arms using the *chi-square* test for proportions and Student’s *t*-test for continuous variables. The primary analysis of outcomes will be a modified per protocol analysis comparing hazards of treatment failure with 95 % confidence intervals in the placebo arm with hazards in the amoxicillin arm. In order for an enrolled child to be included in the modified per protocol analysis, s/he must have received 5 out of 6 doses, including 4 doses in the first two days. Adjusted hazard rate ratios will be estimated accounting for the effect of any baseline variable found to differ between the two arms. Intention to treat analysis will also be performed for comparison.

Additionally, analyses will be done to identify predictors of treatment failure. Baseline characteristics between cumulative treatment success and failure groups by day 3 will be compared using the *chi square* test for categorical variables and Student’s *t*-test for continuous variables. A multivariate model will be constructed to look for determinants of cumulative treatment failure at day 3. All analysis will be done using STATA version 12. Sub-group analyses will be performed by age category, nasopharyngeal carriage, wheezing and initial fever.

Investigators propose to conduct one interim analysis using the O’Brien Fleming rule when 50 % per protocol subject accrual has been achieved. Termination will be done if p < 0.01 is observed with respect to the null hypothesis of inferiority. Final analysis will be done once enrollment is complete.

### Ethical considerations

#### Ethical approval and consent

The study received ethical approval by the Ethics Review Committee of Aga Khan University (ERC reference number 2786-Ped-ERC-13). An ethical opinion was also sought from the Faculty of Medicine Ethics Committee at Southampton University, UK. A written informed consent is sought by an independent physician from the caretakers of all eligible children for participation in the study. All study procedures abide by the approved protocols.

#### Patient safety

**Fig. 3:**
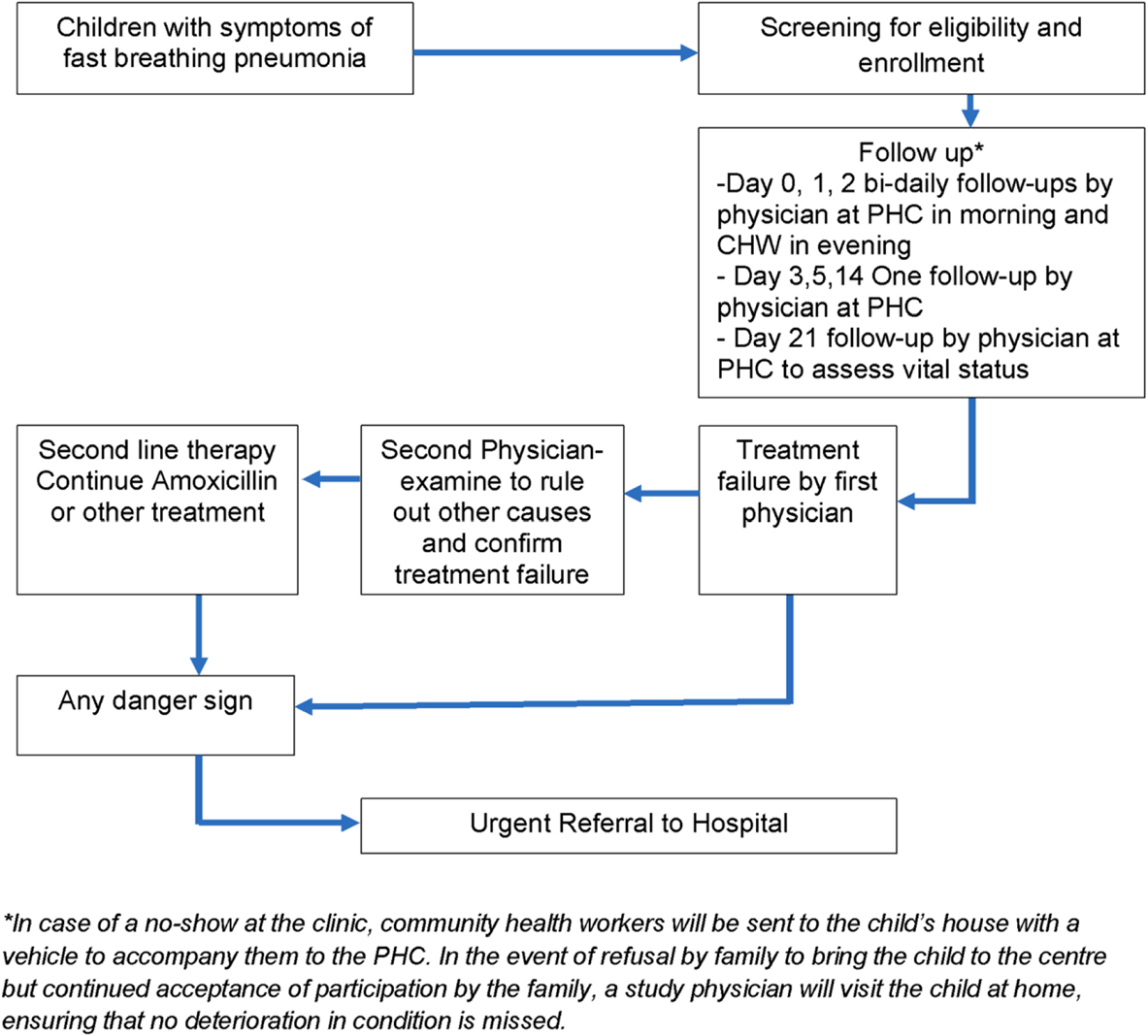
Framework to ensure patient safety in the study

All children enrolled in the trial are closely monitored for deterioration or development of new clinical signs. Children who are sick but not enrolled are followed for safety and ethical reasons, but will not be included in study analyses or reporting. There are several safety nets to ensure that there is quick identification of both early as well as late deterioration with immediate referral and treatment (Fig. 3).

A trial steering committee, including international experts appointed by study sponsors, provides overall supervision of the safety and wellbeing of participants and ensures adherence to standards of the Medical Research Council. An independent Data Safety Monitoring Board (DSMB) has been formed to monitor safety of study participants, providing study oversight by un-blinded monitoring of study results to ensure safety of participants. If a safety signal is observed, the DSMB may stop this study prior to full recruitment. It is possible that placebo and amoxicillin are not similar in the management of fast breathing pneumonia and that the participants receiving placebo suffer a higher failure rate. Investigators believe that the existence of safety net will minimize the chances of harm to participants.

#### Possible risks

The main risk in this group of children is treatment failure, which can be as severe as death. Investigators believe that there is equipoise regarding utility of antibiotics for mild lower respiratory illness. They agree that pneumonia is an important cause of mortality beyond neonatal age. However “non-severe pneumonia” defined as isolated fast breathing in the absence of danger signs is highly unlikely to lead to mortality because non-severe (fast breathing) pneumonia is primarily viral in origin and antibiotic therapy is unnecessary for this syndrome. Death is extremely unlikely as the case-fatality rate previously reported with non-severe (fast breathing) pneumonia is negligible [28]. Fatality is known to be high only in areas of severe malnutrition and with high HIV prevalence (8–10). In comparison the proposed area of the study is a low HIV setting [30]. Similarly, children with danger signs indicating very severe disease will not be enrolled and will be provided facilitated referral to a hospital. Children having weight-for-height/length less than −3 standard deviations will be considered eligible only if they have no other exclusion signs including pedal edema and danger signs. Those needing hospitalization will be transported to tertiary care hospital facility and will not be enrolled. Nutritional counseling and follow-up will be done for the malnourished.

There is a potential hazard for viral infections to be subsequently followed by bacterial infections with an elevated mortality risk beyond two weeks for pneumonia. Therefore, a status check at day 21 will be done. Moreover, there will be ongoing surveillance and a reporting system for adverse events (AEs). All major severe adverse events (SAEs): anaphylaxis, organ failure and death will be promptly recorded on the adverse event reporting form and relayed to the DSMB.

## Discussion

### Limitations and bias

Potential sources of bias are listed below with descriptions of the procedures in place to overcome each.

1. Selection Bias: Allocation sequence is generated by a person independent to the trial and is done electronically via computer using random selection method. Block randomization technique with varied size of block in order to ensure allocation sequence concealment is used. The code list identifying patient with the allocation (drug or placebo) is kept secret until after analysis or until the recommendations of the data safety and monitoring board (DSMB).
2. Performance bias: This is a blinded study in which the drug assignment will be concealed from patients, parents, and study personnel. To ensure blinding, self-adhesive, pre-coded sticking labels with the unique identification numbers will be prepared at the CTU and applied to the medication bottles before providing to the study physicians. This will minimize the chance of systematic differences between groups in the care that is provided. Drug will be dispensed by trained community health care workers who are unrelated to the randomization and outcome assessment processes.
3. Detection bias: This is a blinded study in which the drug assignment is concealed from study personnel who are randomising and assessing the outcome. Randomization codes are kept safe with a person completely unrelated with the trial and broken only after analysis or upon the recommendation of the DSMB. This will ensure blinding of both the allocating physician as well as the one performing outcome assessment.
4. Attrition bias: The period of follow-up is short and loss to follow-up in the study area is minimal. All efforts will be made to ensure follow-up in the trial. In case of a no-show at the clinic (either for receiving the study medication or for follow-up), community health workers will be sent to the baby’s house with a vehicle to accompany them to the PHC centres. In the event of refusal by family to bring the baby to the centre but continued acceptance of participation by the family, a study physician will visit the child at home, to give treatment and assess outcomes.
5. Reporting bias: All results, whether negative or positive will be explicitly reported in any manuscript that results from the study.
6. Non-compliance bias: Non-compliance could be due to adverse effects or due to seeking care elsewhere, either migration. However, as the therapy duration is short, effects of any migration are expected to be minimal. Based on prior experience on these field sites compliance rates exceed 93 %. Previous published trials on pneumonia in the region have also shown >96 % adherence with the study protocol. Based on the previous experience of investigators they will ensure trial compliance of 95 % of the participants, which will be considered as one of the benchmarks of trial quality. Moreover, from same experience from these sites, only 3 % of enrolled subjects seek therapy from elsewhere while enrolled in our study. In the proposed trial investigators will seek information from the family daily on whether outside therapy with antibiotics has been given, and also examine any reported therapy during the home visit.
7. Therapeutic misconception: Research participants with active disease are more likely to believe that they will benefit from participation in the trial. For this purpose an independent physician has been kept to obtain consent independent of the study physician and other PHC activities.

### Dissemination

Dissemination and promotion of study results will happen through small- and medium-scale communication events with stakeholders as well as symposia, publications, and websites that will favor the exchange of ideas. The results will benefit academic communities, health officials, clinicians, care providers, researchers, and population at large regarding usefulness of antibiotic in management of fast breathing pneumonia in children two months to five years old.

### Conclusion

If the trial shows non-inferiority of placebo as compared to amoxicillin, the trial data may make the case for changing treatment guidelines and policy for fast breathing pneumonia. The trial results will strengthen the evidence base to re-consider the WHO guidelines for management of pneumonia with prudent use of antibiotics. Findings will be generalizable to resource limited settings with low HIV prevalence and malaria endemic countries with Hib and Pneumococcal vaccines in their national immunization plan. There will be significant economic implications and consequences for the global problem of emerging antibiotic resistance, for constrained health systems with high burden of infant and children deaths and for families who pay their expenditures out-of-pocket.

### Availability of data and materials

Data will available on public domain 2 years after the publication of the main manuscripts. Processes will be developed to facilitate data sharing for scientific utilization in a collaborative manner.

## Abbreviations

WHO: World Health Organization
RCT: Randomized Controlled Trial
TF: Treatment Failure
PHC: Primary Health Center
CHW: Community Health Worker
CTU: Clinical Trial Unit
TSC: Trial Steering Committee
DSMB: Data Safety and Monitoring Board
AKU: Aga Khan University
CRF: Case Report Form
SOP: Standard Operating Procedure
AERF: Adverse event reporting form
SAE: Severe Adverse Event
